# SNPInterForest: A new method for detecting epistatic interactions

**DOI:** 10.1186/1471-2105-12-469

**Published:** 2011-12-12

**Authors:** Makiko Yoshida, Asako Koike

**Affiliations:** 1 Central Research Laboratory, Hitachi, Ltd., Higashi-Koigakubo, Kokubunji-shi, Tokyo, Japan

## Abstract

**Background:**

Multiple genetic factors and their interactive effects are speculated to contribute to complex diseases. Detecting such genetic interactive effects, i.e., epistatic interactions, however, remains a significant challenge in large-scale association studies.

**Results:**

We have developed a new method, named SNPInterForest, for identifying epistatic interactions by extending an ensemble learning technique called random forest. Random forest is a predictive method that has been proposed for use in discovering single-nucleotide polymorphisms (SNPs), which are most predictive of the disease status in association studies. However, it is less sensitive to SNPs with little marginal effect. Furthermore, it does not natively exhibit information on interaction patterns of susceptibility SNPs. We extended the random forest framework to overcome the above limitations by means of (i) modifying the construction of the random forest and (ii) implementing a procedure for extracting interaction patterns from the constructed random forest. The performance of the proposed method was evaluated by simulated data under a wide spectrum of disease models. SNPInterForest performed very well in successfully identifying pure epistatic interactions with high precision and was still more than capable of concurrently identifying multiple interactions under the existence of genetic heterogeneity. It was also performed on real GWAS data of rheumatoid arthritis from the Wellcome Trust Case Control Consortium (WTCCC), and novel potential interactions were reported.

**Conclusions:**

SNPInterForest, offering an efficient means to detect epistatic interactions without statistical analyses, is promising for practical use as a way to reveal the epistatic interactions involved in common complex diseases.

## Background

The identification and characterization of susceptibility gene variations relevant to common complex diseases is one of the central goals of human genetics. Recent advances in genetic technology, such as high-throughput genotyping techniques based on microarrays, have presented not only substantial opportunities but also unprecedented challenges for resolving the genetic architecture of complex diseases. For example, genome-wide association studies (GWAS) have recently been established, in which a huge number of single-nucleotide polymorphisms (SNPs) across the whole genome are examined in a sample of cases and controls to determine which SNPs are associated with a specific disease [1]. While GWAS have already achieved a certain degree of success in newly detecting disease-associated SNPs, a large proportion of the genetic factors involved in complex diseases has yet to be uncovered. This might be partly because current GWAS have primarily focused on testing association of only a single SNP at a time. Because of the sophisticated regulatory mechanisms encoded in the human genome, complex diseases are speculated to be caused by multiple variations and their interactive effects, which are referred to as epistatic interactions. These variations possibly contribute to a certain disease only by pure interactions whereas they may show little effect individually. Such variations would not be detected in single-variation association analyses. Identifying the epistatic interactions will therefore likely be key to further understanding the pathogenesis of complex diseases.

Identifying epistatic interactions between multiple SNPs remains both statistically and computationally challenging in large-scale association studies. The challenges include the high-dimensionality problem, computational capability, the absence of marginal effects, the multiple testing problem, and genetic heterogeneity. Some computational methods have been proposed to address this problem, other than traditional parametric statistical methods such as logistic regression. They can be roughly divided into three categories on the basis of their optimization strategies: brute-force search methods, greedy search methods, and stochastic search methods. Brute-force search methods, such as the multifactor-dimensionality reduction (MDR; [2]), basically rely heavily on exhaustively verifying all possible SNP combinations. Greedy search methods, such as the set association approach [3], select SNPs one by one such that selected SNP combinations have maximum interactive effects. Since the first SNP is selected on the basis of a univariate test, these methods will fail to detect SNP combinations when marginal effects of each SNP are weak or absent, even if they do have effects caused by interaction. Most methods suffer from the multiple testing problem. Adjusting for multiple testing results in a decrease in power to detect weaker associations of susceptibility SNPs. Furthermore, an additional concern that previous methods have confronted is the presence of genetic heterogeneity; that is, there might be multiple different genetic factors that are independently associated with the same disease. Methods that lump an entire sample into a single group to assess the average effects will likely degrade their power to detect associations.

Recently, interest has grown in applying machine learning techniques to analyzing genetic association studies due to their ability to thrive on large-scale and high-dimensional data. In one recent study, Bayesian epistasis association mapping (BEAM; [4]) designed a form of Bayesian model selection and used a Markov chain Monte Carlo sampling strategy to estimate the model parameters. A recent approach called SNPHarvester [5] searches for SNP groups with significant associations on the basis of an enhanced variation of the genetic programming algorithm. These methods perform reasonably in certain model cases. However, not all the concerns have been fully addressed: the detection power of susceptibility SNPs is still not sufficient and the effectiveness in the presence of genetic heterogeneity has not been validated; in addition, they all partly utilize statistical association tests with adjustments for multiple testing applied. Thus, further high-performance methods are still desired from different perspectives.

In this study, to identify epistatic interactions in large-scale association studies, we propose a new method that uses an ensemble learning technique called random forest [6]. The random forest technique is a tree-based predictive method, which produces a series of classification trees using a large set of predictor variables. It was proposed as a way to discover the SNPs that are most predictive of the disease status in large-scale association studies (e.g. [7,8]). The technique can detect SNPs that are likely to affect disease susceptibility from among a large number of SNPs, taking into account the effects of interactions among them at some level. It has, however, some limitations for identifying epistatic interactions. First, it may perform poorly in detecting SNPs that have little marginal effect. Furthermore, it does not natively exhibit information on interaction patterns of susceptibility SNPs. We extend the random forest framework to overcome the limitations and establish an applicable method for identifying epistatic interactions. Having been developed with all the above-mentioned concerns taken into consideration, this method avoids many of the difficulties previous approaches have faced and proves to be very effective in a wide range of simulations reflecting practical disease models that incorporate the absence of marginal effects and also genetic heterogeneity.

## Results and discussion

### Analysis using simulated data

#### Models of epistatic interactions with weak marginal effects

We considered three disease models with different characteristics: an additive model that contains two SNPs that independently contribute to the disease risk, a multiplicative model that involves interactions of two SNPs with multiplicative effects, and a threshold model that involves interactions of two SNPs with threshold effects. The marginal effect size was set to λ = 0.5. For *r^2^*, two values (1.0 and 0.7) were used for data simulation; for the MAF, four values (0.05, 0.1, 0.2, and 0.5) were considered. There were thus eight parameter settings for each disease model.

**Figure 1:**
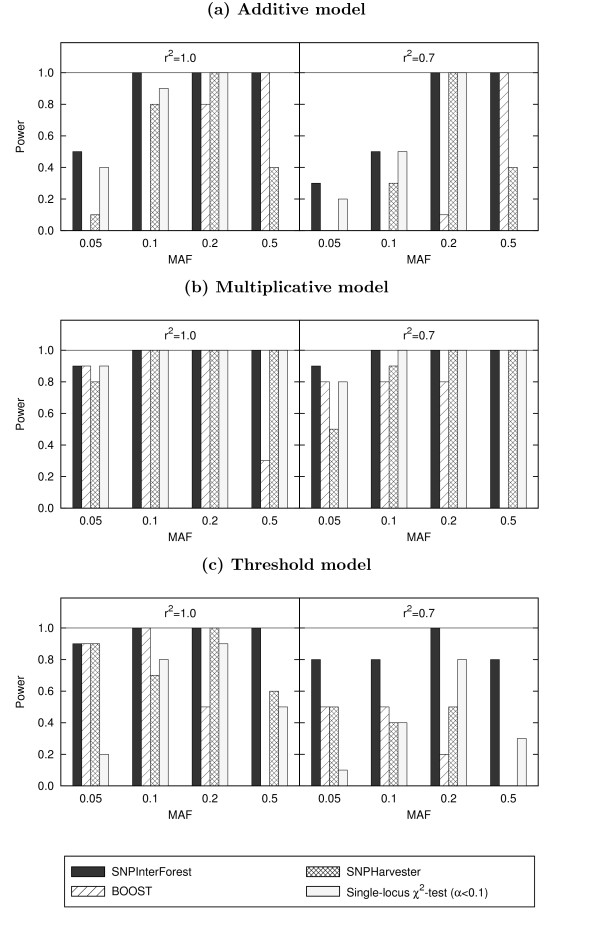
**Performance for models of epistatic interactions with weak marginal effects**. Performance of SNPInterForest compared with those of BOOST and SNPHarvester for models of epistatic interactions with weak marginal effects: (a) additive model, (b) multiplicative model, and (c) threshold model.

By using SNPInterForest, the importance of the SNPs concerned was found to be clearly distinguishable from that of the others by the importance score derived in this method. Besides, the interaction score using the number of simultaneous appearances in tree branches in the random forest is shown to be able to correctly identify the interaction of these SNPs. Figure 1 compares the simulation results of SNPInterForest with those of BOOST [9] and SNPHarvester [5]. For reference, power of the single-locus chi-square test to detect both loci in the interaction with a rather liberal threshold of 0.1 (Bonferroni corrected) is also given in the figure. The results show that SNPInterForest outperforms both BOOST and SNPHarvester for all three models. In practice, the performance of BOOST is significantly low in the cases with low MAF for the additive model, and in the cases with high MAF for the multiplicative and threshold models. As discussed in [10], MAF at disease-associated SNPs strongly influences the detectability of those SNPs. Since BOOST is based on statistical tests through an exhaustive search strategy, correction for multiple testing might become a severe burden where multilocus information is not so large as to negate its cost; the interaction-based search involves 10^3 ^times as many tests as the single-locus search does. SNPHarvester also utilizes in part statistical association tests. On the other hand, SNPInterForest achieves better or even considerably higher performance even in these parameter settings. As for precision, SNPInterForest and SNPHarvester do not detect any spurious associations, whereas BOOST falsely detects five interactions among these datasets.

#### Models of pure epistatic interactions without marginal effects

First of all, we studied simple models where two SNPs are involved in the etiology of disease by a pure epistatic interaction; these are the original models of Velez *et al*. [11]. Here, six models with the parameters set to practical values were considered (*h^2 ^*ranged from 0.2 to 0.4, and the MAFs were 0.2 and 0.4).

**Figure 2:**
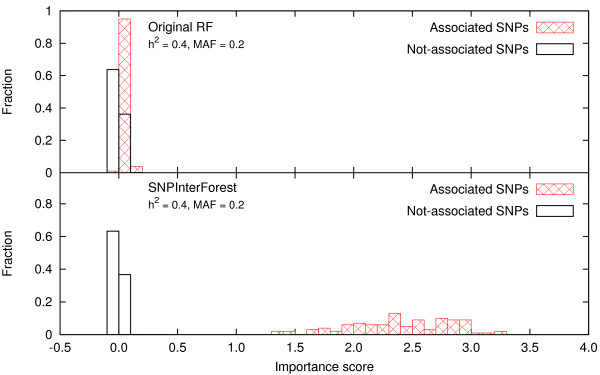
**Distribution of the importance score compared with the original RF**. Distribution of the importance score which is computed by permutation testing for a simple model of pure epistatic interactions. The upper panel shows the results from the original random forest, and the lower panel shows the result from SNPInterForest. The SNPs associated with disease are represented by red boxes, and the other SNPs are represented by black boxes.

Figure 2 shows the distribution of the importance score for the SNPs associated with disease and that of the other SNPs. Compared with the original random forest, the ability to detect SNPs that are associated with disease by only a pure epistatic interaction is significantly improved in this method by means of multiple-SNP selection at each node in the construction of the random forest; the original random forest where only a single SNP is used fails to detect the significance of these SNPs.

Table 1 summarizes a comparison of the number of interacting SNP pairs detected in the simulation by SNPInterForest, BOOST, and SNPHarvester. The results show that the power of the SNPInterForest and BOOST is superior to that of SNPHarvester for all models considered. In fact, both methods work very well to identify successfully almost 100 percent of the epistatic interactions for all models, while SNPHarvester succeeds in only 70-80 percent over all models. We note that the original random forest identifies no interaction for models with *h^2 ^*of 0.2 and MAF of 0.4, and succeeds in at most a few percent of the datasets for models with high heritability. The single-locus chi-square test also loses its power completely for these models as is naturally expected from the nature of the models. On the other hand, BOOST is shown to suffer from false positives over all models, while false discovery rates for SNPInterForest and SNPHarvester are quite low.

**Table 1:** Comparison of performances of different methods on simple models of pure epistatic interactions

	SNPInterForest		BOOST		SNPHarvester	
	# of true positives (N_total _= 50)	# of false positives	# of true positives (N_total _= 50)	# of false positives	# of true positives (N_total _= 50)	# of false positives
h^2 ^= 0.4, MAF = 0.2	50	0	50	6	37	0
h^2 ^= 0.4, MAF = 0.4	50	0	50	9	38	0
h^2 ^= 0.3, MAF = 0.2	50	0	50	1	36	0
h^2 ^= 0.3, MAF = 0.4	50	0	50	5	38	0
h^2 ^= 0.2, MAF = 0.2	49	1	50	1	36	0
h^2 ^= 0.2, MAF = 0.4	49	0	50	5	39	1

	# of true positives (N_total _= 5)	# of false positives	# of true positives (N_total _= 5)	# of false positives	# of true positives (N_total _= 5)	# of false positives

3SNP interaction	5	0	-	-	0	0

The proposed method was further examined to test whether it can identify interactions involving more than two SNPs. For this purpose, we considered a disease model that contained three SNPs interactively associated with disease: two that are moderately associated with disease by a pure epistatic interaction and another with a weaker effect that amplifies the interactive effect in collaboration with the other two. Five simulated datasets reproducing this model were constructed by redesigning the above two-SNP interaction model for moderate association (*h^2 ^*of 0.2 and the MAF of 0.4) such that one of the non-associated SNPs was realigned to interactively affect disease susceptibility with the two associated SNPs. The interaction score was computed for combinations consisting of three SNPs. The three interacting SNPs are successfully captured by the interaction score for all five datasets (Table 1). The results suggest that the proposed method can be directly generalized to identify higher-order interactions. These interactions are not identified by SNPHarvester. BOOST cannot identify interactions with more than two SNPs.

Second, we explored the case where multiple epistatic interactions affect disease susceptibility, which is likely to happen especially in the pathogenesis of complex diseases. Here, we considered six hybrid models, which included five interactions each involving two SNPs. For each hybrid model, we constructed simulated datasets by mixing five datasets from each of the above single two-SNP interaction models, respectively (the same as in the work of Yang *et al*. [5]). Furthermore, we extended the simulation studies to incorporate genetic heterogeneity. To mimic heterogeneous causal interactions, we constructed simulated datasets for heterogeneous models again using the single two-SNP interaction models, but in this case by simply combining two datasets from each of the models. That comes to six heterogeneous models containing 400 cases and 400 controls, which include two interactions that independently predispose respective halves of the patients to the same disease.

It was found that SNPInterForest can simultaneously detect SNPs that affect disease susceptibility in multiple ways. Of particular importance is that it can automatically identify the interacting SNP combinations by the interaction score. Tables 2 and 3 summarize a comparison of the number of interacting SNP pairs detected in the simulation by SNPInterForest, BOOST, and SNPHarvester for hybrid models and heterogeneous models, respectively. SNPHarvester identifies only a portion of the multiple interacting SNP pairs in each dataset. BOOST considerably outperforms SNPHarvester. However, it also suffers to some extent from false positives. SNPInterForest performs comparably to BOOST in most cases, keeping false discovery rates lower. The results suggest that the new method can effectively contribute to concurrently identifying multiple interactions, even under the existence of genetic heterogeneity.

**Table 2:** Comparison of performances of different methods on hybrid models

	SNPInterForest		BOOST		SNPHarvester	
	# of true positives (N_total _= 250)	# of false positives	# of true positives (N_total _= 250)	# of false positives	# of true positives (N_total _= 250)	# of false positives
h^2 ^= 0.4, MAF = 0.2	250	0	250	10	119	0
h^2 ^= 0.4, MAF = 0.4	250	0	250	3	115	0
h^2 ^= 0.3, MAF = 0.2	250	0	250	7	103	0
h^2 ^= 0.3, MAF = 0.4	250	0	250	8	108	0
h^2 ^= 0.2, MAF = 0.2	249	2	250	9	111	1
h^2 ^= 0.2, MAF = 0.4	248	1	250	2	102	0

**Table 3:** Comparison of performances of different methods on heterogeneous models

	SNPInterForest		BOOST		SNPHarvester	
	# of true positives (N_total _= 100)	# of false positives	# of true positives (N_total _= 100)	# of false positives	# of true positives (N_total _= 100)	# of false positives
h^2 ^= 0.4, MAF = 0.2	98	1	98	5	64	0
h^2 ^= 0.4, MAF = 0.4	98	0	100	7	66	1
h^2 ^= 0.3, MAF = 0.2	90	1	81	6	59	0
h^2 ^= 0.3, MAF = 0.4	80	0	100	7	62	0
h^2 ^= 0.2, MAF = 0.2	28	4	16	10	19	0
h^2 ^= 0.2, MAF = 0.4	38	1	78	3	29	2

#### False discovery rates evaluation on null simulations

As already mentioned in previous subsections, SNPInterForest is not affected by deleterious rates of spurious detection in contrast to achieving high recall rates. On the other hand, the tradeoff between power and precision has been classical suffering (e.g., [12]). To further approximate the precision, we simulated datasets without any disease-associated SNPs. We constructed 50 datasets containing 1,000 SNP markers genotyped for 2,000 cases and 2,000 controls, where the SNPs were generated independently with MAF uniformly distributed in [0.05, 0.5]. The running parameters for the programs were set in the same way as for the disease models. In the null simulations, SNPInterForest did not report any false positives.

### Analysis using real GWAS data

We performed SNPInterForest on real GWAS data of rheumatoid arthritis (RA) from the Wellcome Trust Case Control Consortium (WTCCC; [13]), which consisted of about 500 K SNPs and 3,499 individuals (1,999 cases and 1,500 controls). The WTCCC distributes two control samples (1958 British Birth Cohort and UK Blood Service Control Group), each of which contains 1,500 individuals. Because the latter sample cannot be accessed by commercial organizations, we used only the former. We narrowed the search down to the top 10,000 SNPs selected by single-locus association study to accommodate computational requirements. It took about 98 hours for SNPInterForest to handle it on a Linux system with a single CPU (CPU: Intel Xeon 2.67 GHz) and a memory of 6 GB. The running time of SNPInterForest, SNPHarvester, and BOOST on this dataset is summarized in Table 4. We note that SNPHarvester requires substantial memory space and its running time is highly sensitive to the size of memory. SNPInterForest identified two novel interactions from this dataset, which are summarized in Table 5. Information on genes related to the SNPs in these interactions is also provided in Table 6. Although biological interpretation of the interacting SNPs is yet unclear, there are some functions in these genes that seem to have possibility to relate with RA, such as inflammatory response in the gene *PROK2*. These interactions were also identified by BOOST. BOOST detected a dozen of other interactions, which were not detected by SNPInterForest.

**Table 4:** Running time of different methods on WTCCC RA data

SNPInterForest	BOOST	SNPHarvester
98 hours	11 min	5 hours

**Table 5:** Interactions identified by SNPInterForest in WTCCC RA data

Interacted SNPs	Location	Type	MAF
rs17665418	3p13	gSNP	Case: 0.12/Control: 0.067
rs2121526	10q21.1	iSNP	Case: 0.12/Control: 0.057

rs17665418	3p13	gSNP	Case: 0.12/Control: 0.067
rs4799934	18q12.2	iSNP	Case: 0.12/Control: 0.056

**Table 6:** Gene information related to the SNPs identified in WTCCC RA data

SNPs	Closest genes	Classification for the genes (morceaux)
rs17665418	*PROK2*	G-protein coupled receptor protein signaling pathway Angiogenesis, Cell proliferation, Chemotaxis Inflammatory response Positive regulation of smooth muscle contraction Sensory perception of pain
rs2121526	*PCDH15*	Cell adhesion Sensory perception of light stimulus
rs4799934	*BRUNOL4*	Embryo development Germ cell development mRNA splice site selection

## Conclusions

We proposed a new effective method, named SNPInterForest, for identifying epistatic interactions in large-scale association studies by extending an ensemble learning technique called random forest. SNPInterForest, which introduces a new procedure to capture SNP interactions on the basis of the tree structure in the random forest and modifies the construction method of a random forest, offers an efficient means to detect epistatic interactions without statistical analyses. Although it is relatively computationally expensive, the effectiveness of the proposed method was verified in extensive simulation studies under a wide spectrum of disease models. This method achieved considerable improvements compared to the original random forest in identifying pure epistatic interactions, and outperformed the existing methods in high recall rates while keeping low false discovery rates. Encouraged by the success in detecting multiple SNP interactions, we plan to extend our current method in the future to infer the overall genetic network involved in the pathogenesis of complex diseases. The software is available at: https://gwas.lifesciencedb.jp/SNPInterForest/index.html.

## Methods

### Random forest

The random forest technique is an ensemble learning technique for conducting classification analyses [6]. In brief, it constructs a collection of classification trees to aggregate them into one robust classifier, where each of the trees is built with two stochastic modifications applied to the deterministic tree-growing algorithm: (i) each tree is trained on a bootstrap sample of the original sample; (ii) at each node in the trees, the best split is chosen from among a randomly selected subset of the predictor variables instead of the full set. A desirable feature of the random forest technique is that it provides an importance score for respective variables that measures their contribution to the predictive accuracy. The contribution of a variable is quantified via permutation testing by disrupting the dependence between the variable and the class and measuring how much the prediction accuracy of the trees decreases compared to that in the original setting. Specifically, the importance score of a certain variable is defined as follows [14]. For individual i, let X_i _represent the vector of variable values, y_i _its true class, V_j_(X_i_) the prediction of tree j, and t_ij _an indicator taking value 1 when individual i is out-of-bag for the bootstrap sample of tree j and 0 otherwise. Let X^(A, j) ^represent the vector of variable values with the value of variable A randomly permuted among the out-of-bag individuals for tree j. Letting F(C) denote the indicator function taking value 1 when the condition C is true and 0 otherwise, the importance score of a variable A is calculated as follows:

(1) $$\text{I}\left(\text{A}\right)=\frac{1}{\text{T}}\overset{\text{T}}{\underset{\text{j}=1}{\sum }}\frac{1}{{\text{N}}_{\text{j}}}\overset{\text{N}}{\underset{\text{i}=1}{\sum }}\left[\text{F}\left({\text{V}}_{\text{j}}\left({\text{X}}_{\text{i}}\right)={\text{y}}_{\text{i}}\right)-\text{F}\left({\text{V}}_{\text{j}}\left(X_{\text{i}}^{\left(\text{A},\;\text{j}\right)}\right)={\text{y}}_{\text{i}}\right)\right]{\text{t}}_{\text{ij}},$$

where N_j _represents the number of out-of-bag individuals for tree j, N is the total number of individuals in the sample, and T is the total number of trees. Importantly, the importance score encompasses the effects of interactions among variables, as it assesses the contribution of a certain variable to the prediction in the presence of all other variables. In the context of genetic association studies, this importance score can be used to discover variables, i. e., SNPs, that are most predictive of the disease status and thereby likely to affect disease susceptibility [15].

The random forest technique can deal with a large number of SNPs. It is also thought to be easily adaptable to handling genetic heterogeneity, since separate models are automatically fitted to subsets of data defined by early splits in the trees in the course of tree-building processes. However, its critical disadvantage in detecting epistatic interactions is that it is less sensitive for SNPs with little marginal effect. When the contribution of a SNP is caused only by a pure interaction with other SNPs, the importance score of the relevant SNP diminishes. This is a primary limitation that must be overcome to enhance its ability as a detection tool of epistatic interactions. The random forest framework can also be extended from the viewpoint that the tree-based model has good interpretability in terms of representing variable interactions. While the importance score natively provided by the technique can be used to prioritize susceptibility SNPs, it is computed for individual SNPs and does not reveal the combination patterns of interacting SNPs. Further exploration is required to extract the interaction patterns among significant SNPs. In the next section, we describe a means to improve the random forest technique in terms of the absence of marginal effects. Then, rather than a statistical analysis, we introduce a new procedure to capture the SNP interactions on the basis of the tree structure in the random forest.

### SNPInterForest: extension of random forest for detecting epistatic interactions

The new method, named SNPInterForest, is designed in the following way. First, the random forest is constructed on case-control data as a classifier to discriminate between cases and controls with SNPs as categorical variables that have three possible values, i. e., the three genotype values. In this regard, an emphasis is placed on deriving the importance score of each SNP that measures its contribution in determining the disease status. In the construction of classification trees, a sample is recursively split into two subsamples on the basis of a chosen split variable, with each split improving the homogeneity of the sample contained in the present node with regard to the disease status. In this process, the Gini index [16] is used to assess the homogeneity of a sample at a certain node, which is defined for node γ as follows:

(2) $$\varphi \left(\gamma \right)=1-{\text{f}}_{0}^{2}-{\text{f}}_{1}^{2},$$

where f_1 _and f_0 _are the relative frequencies of cases and controls, respectively. Now, for a split at node γ that yields two sub-nodes, γ_1 _and γ_r_, the improvement of homogeneity by this split is calculated by the decrease in the Gini index as follows:

(3) $$\Delta \varphi \left(\gamma \right)=\varphi \left(\gamma \right)-{\text{f}}_{1}\varphi \left(\gamma_{1}\right)-{\text{f}}_{\text{r}}\varphi \left(\gamma_{\text{r}}\right),$$

where f_1 _and f_r _are the fractions of samples in γ that fall into γ_1 _and γ_r_, respectively.

**Figure 3:**
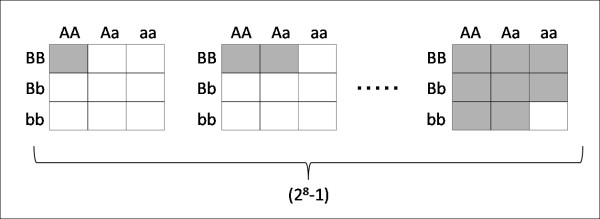
**Illustration of how the best partition is evaluated for a combination of two SNPs**. A combination of two SNPs can have nine possible values (corresponding to respective cells in each panel), and its best partition is chosen from among (2**^8^**-1) ways of splitting rules (corresponding to the number of panels each of which depicts one of the dual-splitting rules by white and grey cells).

Here, the construction of classification trees is modified such that, when choosing a split variable at each node, a combination of multiple SNPs as well as a single SNP is allowed. In the original tree-building manner, the single SNP that provides the best partition, that is, that gives the maximum decrease in the Gini index, is chosen for a split variable. It is suspected that using only a single SNP tends to decrease the possibility to ever appear in trees for SNPs without marginal effects, since the first split variable is selected on the basis of just individual effects. In the modified version, combinations of multiple SNPs are also considered as candidates for a split variable, and the best combination of SNPs or the best single SNP is chosen. When evaluating the best partition by a certain candidate variable, all possible splitting rules are taken into account. Specifically, a candidate variable of a combination of n SNPs can have 3^n ^possible values, and the best partition for it is chosen from among $\left(2^{3^{\text{n}}-1}-1\right)$ ways of splitting rules. Figure 3 illustrates the case for a combination of two SNPs for reference. This modification is expected to prevent the importance scores of SNPs without marginal effects from being underestimated. In this study, combinations of up to two SNPs are considered for one node. We think this is reasonable enough, considering it to be least likely that among SNPs interactively associated with disease, any set of two SNPs do not show any effect at all.

Next, a procedure is implemented for extracting interaction patterns from the constructed random forest. In the random forest, each path from the root node to the leaf node, i. e., a branch, in the classification trees indicates a possible interaction among variables on that branch. In other words, interactions of variables are represented within a branch in trees. Thus, in a specific sense, it may be argued that when a certain combination of SNPs appears more frequently in the same branches, those SNPs should interact more strongly with each other in affecting the disease outcome. Therefore, for each SNP combination, the number of simultaneous appearances in the same branches is counted over all trees in the random forest as a measure of its interaction strength. Specifically, it is defined for a SNP combination G as the summation of the number of appearances of G in a branch b, n_b_(G), for all branches over all trees in the random forest as follows:

(4) $$\text{inter}\left(\text{G}\right)=\overset{\text{T}}{\underset{\text{j}=1}{\sum }}\underset{\text{b}\in {\text{B}}_{\text{j}}}{\sum }{\text{n}}_{\text{b}}\left(\text{G}\right),$$

where B_j _represents the collection of all branches in tree j, and T is the total number of trees. Note that a certain combination is to be counted only once to avoid bias towards those at the top of a branch. This measurement, hereafter referred to as the interaction score, can be used to discover interacting SNP groups. We normalize the interaction score for each SNP combination by using its respective baseline level, which indicates the expected number of simultaneous appearances under the null hypothesis, i. e., the hypothesis that the SNP combinations concerned do not interact. The baseline levels for respective SNP combinations are estimated in the following manner. First, the SNP positions in the trees in the random forest constructed are randomized with the numbers of the respective SNPs and the topology of the trees kept unchanged. Then, for each SNP combination, the number of simultaneous appearances in the same branches is counted in the same way as for the original random forest. The sequence of these processes is repeated 100 times to develop statistical distributions of the baseline levels for respective SNP combinations. The normalized interaction score for a SNP combination G is thus obtained by subtracting the baseline mean and dividing by the baseline standard deviation as follows:

(5) $$\text{INTER}\left(\text{G}\right)=\frac{\text{inter}\left(\text{G}\right)-\bar{\text{inte}{\text{r}}_{random}\left(\text{G}\right)}}{\sigma \left(\text{inte}{\text{r}}_{random}\left(\text{G}\right)\right)},$$

Where inter_*random*_(G) represents the statistical distribution of the baseline level estimated by randomization, and $\bar{\text{inte}{\text{r}}_{random}\left(\text{G}\right)}$ and σ(inter_*random*_(G)) are its mean and standard deviation, respectively. The normalization is expected to work effectively to pick out weaker interactions and to eliminate contamination from spurious interactions involving SNPs with strong single-handed associations. The detection threshold of the normalized interaction score is empirically determined to be above 25σ throughout the experiments on the basis of simulations on various preliminary datasets. In most cases, the scores of disease-associated interactions are much more significant than others: the maximum scores of non-associated interactions lie around 15σ or less.

### Simulations

The performance of SNPInterForest was evaluated by simulations under diverse disease models and compared to those of recent studies (SNPHarvester [5] and the most recent BOOST [9]) as they were found to be very powerful for detecting epistatic interactions in comprehensive comparisons with other methods. Following many previous studies, we considered models of epistatic interactions with weak marginal effects using the three models in the work of Marchini *et al*. [10], which are additive, multiplicative, and threshold models (Section 4.1). In the additive model, a disease-associated allele at each locus increases the odds of disease independently in a multiplicative fashion both within and between two loci; in the multiplicative model, at least one disease-associated allele must be present at each locus for the odds to increase, and each additional disease-associated allele at the loci further increases the odds in a multiplicative fashion; and the threshold model takes the same forms as a multiplicative model in requiring at least one disease-associated allele at both loci, but additional disease-associated alleles do not increase the risk further. The marginal effect is measured in effect size λ (i.e., the size of effect that is expected to be seen separately at each of the locus for an interaction [10]), and the linkage disequilibrium (LD) between the disease-associated SNPs and a genotyped SNP is measured by *r^2^*. We simulated data under different parameter settings of the LD and the minor allele frequencies (MAFs) of the disease-associated SNPs, considering practical concerns for this issue. Furthermore, we conducted extensive simulation for models of pure epistatic interactions without marginal effects to validate the power of the new method in that problematic case (Section 4.2). A wide range of pure interaction models has been discussed in the literature [17]. Following Yang *et al*. [5], we generated synthetic data on the basis of the interaction models given by Velez *et al*. [11]. The parameters considered for data simulation are the broad-sense heritability *h^2 ^*(i.e., the extent to which affection status can be genetically determined [17]) and the MAFs of the disease-associated SNPs. For this case, in addition to simple single-interaction models, we explored multiple-interaction models and models of heterogeneous interactions that mimic genetic heterogeneity.

We constructed datasets each containing 1,000 SNP markers genotyped for 2,000 cases and 2,000 controls for models of epistatic interactions with weak marginal effects, and datasets containing 1,000 SNP markers genotyped for 200 cases and 200 controls for models of pure epistatic interactions unless otherwise noted. For each disease model, 50 datasets were used in the simulation. The performance of the methods for a specific model was measured by the power defined as the ratio of the number of successful identifications to the total number of simulated datasets. False discovery rates were also estimated as the probability of detecting spurious SNP pairs to be epistatic. For SNPInterForest, we determined the detection threshold of the normalized interaction score to be above 25σ throughout the experiments. There are two main tuning parameters for the random forest: *ntree *(the number of classification trees built in the random forest) and *mtry *(the number of randomly selected candidates for a split variable at each node). Taking into account the prediction error and, more particularly, the stability of the estimates of the importance score, we empirically determined their values as *ntree *= 10,000, and *mtry *= 50,000, which is around 10% of the number of all the possible candidates (here, $\left(1,000+{\text{C}}_{1,000}^{2}\right)$ for 1,000 SNPs). In general, the larger the value yields, the better the performance within the range we studied, although *ntree *has minimal effect over a wide range of values on the order of 1,000-10,000 trees particularly in the case of detecting strong associations. The parameter for SNPHarvester was set as *SuccessiveRun *= 50, as suggested in its original simulation study [5]. The significance threshold for BOOST was selected to be 0.1 after the Bonferroni correction, as used in the original study [9].

## Authors' contributions

MY designed the research, carried out the analysis, and prepared the manuscript. AK added critical insight to the research design and provided significant input to the manuscript. All authors read and approved the final manuscript.
